# Evaluation of mechanical properties of anatomically customized fiber posts using E-glass short fiber-reinforced composite to restore weakened endodontically treated premolars

**DOI:** 10.1186/s12903-024-04102-2

**Published:** 2024-03-11

**Authors:** Dawood Salman Dawood Alshetiwi, Nor Aidaniza Abdul Muttlib, Hatem M. El-Damanhoury, Rabihah Alawi, Normastura Abd Rahman, Nesrin Aly Elsahn, Mohmed Isaqali Karobari

**Affiliations:** 1 Post-graduate Program in Dentistry, School of Dental Sciences, Universiti Sains Malaysia, Health Campus, Kubang Kerian, Kelantan Malaysia; 2 Department of Oral and Craniofacial Health Sciences, College of Dental Medicine, University of Sharjah, Sharjah, United Arab Emirates; 3 Research Institute for Medical and Health Sciences, University of Sharjah, Sharjah, United Arab Emirates; 4 Prosthodontics Unit, School of Dental Sciences, Universiti Sains Malaysia, Health Campus, Kubang Kerian, Kelantan Malaysia; 5 Department of Preventive and Restorative Dentistry, College of Dental Medicine, University of Sharjah, Sharjah, United Arab Emirates; 6 Conservative Dentistry Unit, School of Dental Sciences, Universiti Sains Malaysia, Health Campus, Kubang Kerian, Kelantan Malaysia; 7 Dental Public Health Unit, School of Dental Sciences, Universiti Sains Malaysia, Health Campus, Kubang Kerian, Kelantan Malaysia; 8 Department of Clinical Sciences, College of Dentistry, Ajman University, Ajman, United Arab Emirates; 9 Department of Operative Dentistry, Faculty of Dentistry, Cairo University, Cairo, Egypt; 10 Dental Research Unit, Center for Global health Research, Saveetha Medical College and Hospitals, Institute of Medical and Technical Sciences, Saveetha University, Chennai, 600077 Tamil Nadu India; 11 Department of Restorative Dentistry & Endodontics, Faculty of Dentistry, University of Puthisastra, Phnom Penh 12211, Phnom Penh, Cambodia

**Keywords:** Endodontically treated teeth, Post and core, Fiber-reinforced composite Post, Short fiber-Reinforced composite

## Abstract

**Objective:**

This study was conducted to assess the influence of combining different forms of fiber-reinforced composites (FRC) on the mechanical behavior and bond strength of compromised endodontically treated teeth (ETT).

**Materials and methods:**

Eighty extracted human premolar teeth were randomly divided into five experimental groups according to the type of intra-radicular restoration and the canal preparation design which was either non-flared (Group 1), flared (Groups 2–5), closed-apex (Groups 1,3,5) or open-apex (Groups 2,4). Standard prefabricated fiber posts were used as intra-radicular restoration for Groups 1–3 while Groups 4–5 were restored with anatomically customized relined fiber posts. After composite core fabrication, all samples were sent for an artificial aging process. Fracture resistance and push-out bond strength tests were then carried out through a universal testing machine followed by mode of failure analysis via a stereomicroscope and scanning electron microscope.

**Results:**

Pairwise Log-Rank comparisons revealed that the survival rate of Group 2 and Group 3 was significantly lower than all other groups after artificial aging. The highest fracture resistance value (1796 N) was recorded in Group 5 and was significantly higher than that of the other groups (*p* < 0.05), while Group 2 exhibited the lowest fracture resistance (758 N), which was significantly lower compared to the other groups. Group 5 and Group 4 demonstrated a significantly higher push-out bond strength, at all root thirds, than Group 3, Group 2, and Group 1 (*p* < 0.05). The most frequently observed failure mode in the tested groups occurred between the resin cement and radicular dentin.

**Conclusion:**

The use of short fiber-reinforced composite (SFRC) to reline the prefabricated FRC post has been proven to have superior fracture resistance with favorable failure patterns and increased push-out bond strength values compared to standard prefabricated FRC posts.

## Introduction

The introduction of carbon or glass fiber post systems since the 1990s provided an alternative to cast or prefabricated metallic posts to overcome the challenges of restoring damaged endodontically treated teeth (ETT). Furthermore, these post systems have been developed with a modulus of elasticity closer to that of human dentin, thus, improving the stress distribution along the root and reducing catastrophic failures than the previous metallic posts [1, 2].

Excessive removal of the radicular and coronal dentinal tissue occasionally occurs during endodontic procedures, such as over-flaring canals or attaining straight-line access. Additionally, several clinical situations present with flared and wide root canal space, for instance, endodontic retreatment cases and immature permanent teeth with open apex, thus, requiring intra-radicular posts for core retention [3]. Moreover, the adaptation was compromised due to the discrepancy between the shape of the root canal space and the prefabricated post shape. This inconsistency will result in an excessively thick layer of cement between the post and the radicular dentine. Thus, increasing polymerization shrinkage at the post-dentin interface which could lead to bubble formation and debonding/adhesive failure [4].

Recent studies have proposed an alternative clinical technique to restore structurally-compromised ETT resulting from caries, trauma or congenitally malformed teeth, known as the anatomically customised or relined fiber-reinforced composite (FRC) posts [4, 5]. This technique involves relining the prefabricated FRC post using either conventional [4] or bulk-fill resin composites [6] to enhance adaptation on the walls of non-circular root canals. The adapation was improved by fabricating anatomically-customised fibre posts that conform to the anatomy and taper of the root canal, thus limiting the thickness of the resin cement [7–9].

Recently introduced bulk-fill flowable, discontinuous short fiber-reinforced composite (SFRC) offers advantages and unique properties in restoring large posterior cavities of ETT cavities. For instance, SFRC exhibited superior fracture toughness, flexural strength, and modulus compared to conventional resin composite [10]. Furthermore, SFRC possesses specific properties, such as termination of crack propagation, lower polymerization shrinkage stress, reduced microgap formation, and microleakage compared to existing bulk-fill and conventional resin composites [10, 11]. The application of SFRC inside the root canal as a post-core material was recommended [11, 12]. but only very few studies investigated the mechanical behaivour of prefabricated FRC post relined with SFRC used for restoration of weakened root canal [13–15]. Therefore, this study aimed to investigate the survival, fracture resistance and push-out bond strength after cyclic simultaneous thermomechanical aging of anatomically customized FRC post relined with bulk-fill, flowable version of SFRC and compare the performance with the standard FRC post in restoring flared, immature and compromised root canals. Two null hypotheses were tested in this study as follows (1) There is no significant difference in survival after artificial aging and fracture resistance between standard non-customized fiber posts used to restore ETT, and customized anatomical fiber posts (2) There is no significant difference in push-out bond strength between standard non-customized fiber posts and customized anatomical fiber posts.

## Materials and methods

Ethical approval was obtained from the University of Sharjah – Research Ethics Committee (Ref no. REC-21-09-20-02-S) and Human Research Ethics Committee of Universiti Sains Malaysia (JEPeM-USM) (USM/JEPeM/21,090,637). Eighty extracted human premolar teeth with the root dimensions: root length = 15–16 mm, bucco-lingual dimension at the cervical area = 7–8 mm, and mesio-distal dimension at the cervical area = 5–6 mm were collected from the University Dental Hospital of Sharjah (UDHS).

### Sample preparation

**Fig. 1:**
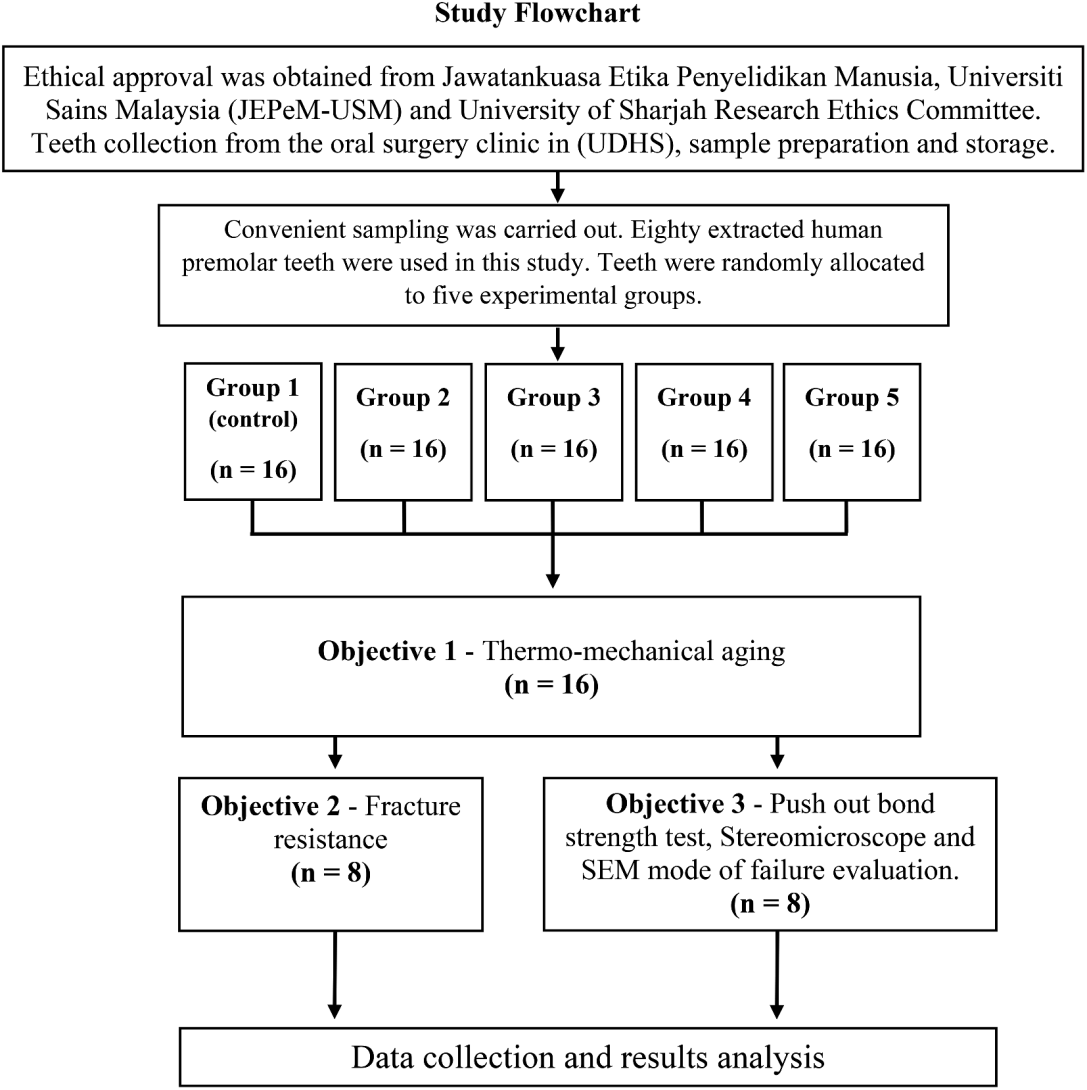
Flowchart of the study design

The extracted teeth were cleaned using an ultrasonic scaler (Dentsply Sirona – MENA, Middle East and North Africa) and sectioned using a low-speed diamond saw (IsoMet 1000, Buehler Ltd., IL, USA) 2 mm above the cemento-enamel junction (CEJ) under water cooling. The working length was determined using a K-file size 10, which was inserted until it appeared at the apex, followed by subtracting 0.5 mm from it [16, 17]. Teeth were then randomly allocated to 5 different groups (Table 1) with sixteen teeth per group (*n* = 16). After artificial aging, eight teeth from each group (*n* = 8) were subjected to fracture resistance test. The remaining eight teeth (*n* = 8) were prepared for push-out bond strength test as displayed in Fig. 1.

For Group 1, Group 3, and Group 5, the sectioned teeth were embedded up to a level of 2 mm short of the CEJ in self-cure acrylic resin (Vertex-Dental, The Netherlands), poured into a cuboidal shape mold, and allowed to set. RelyX™ post drills (3 M Oral Care, St. Paul, MN, United States) sizes white–yellow–red–blue, were used sequentially for simulation of flared root canals (Group 3 and 5), as illustrated in Table 1. The open apex simulation for groups 2 and 4 was performed using Peeso reamers size #1 - #6 (Roydent, USA) to prepare past the apex of the teeth under water cooling along the long axis of the tooth before embedding the roots in the self-cure acrylic resin blocks. This was followed by flared canal simulation as described previously for groups 3 and 5.

Root canal treatment was then carried out in all groups. The WaveOne Gold™ reciprocating file system (Dentsply Sirona – MENA, Middle East and North Africa) was used to prepare the root canal space; a primary (red) or large (white) file was used according to the predetermined canal size and working length. Obturation was performed using WaveOne Gold™ gutta-percha (Dentsply Sirona – MENA) and AH Plus resin sealer (Dentsply Sirona – MENA) via the single cone technique for Groups 1, 3, and 5. On the other hand, the master cone was cut from the apical part and adjusted to the working length until tug-back was evident for Groups 2 and 4 with open apex, followed by obturation similar to the previous groups. The canals’ orifices were temporarily sealed with conventional glass ionomer liner. The teeth were then stored in distilled water for 72 h. Subsequently, the post space was prepared by removing the root canal filling using Lexicon™ gates glidden drills (Dentsply Sirona – MENA) and RelyX™ universal drill (3 M Oral Care, St. Paul, MN, United States), thus, leaving a 5 mm apical seal.

The RelyX™ fiber post drills (3 M Oral Care, St. Paul, MN, United States) in white–yellow–red–blue, were used again sequentially for post-space preparation to obtain a standardized flare and smear layer of the root canals for Groups 2–5. Conversely, the post drills White – Yellow were used only for Group 1. The fiber post length was standardized at three-quarters of the root length of each specimen [18].

**Table 1:** Overview of the experimental groups

Group	Description	Preparation	Materials for root canal restoration
1 (*n* = 16)	*Non-flared, closed apex root canals*	White-Yellow RelyX™ post drills.	(FRC) post (RelyX™, 3 M Oral Care, St. Paul, MN, United States)
2 (*n* = 16)	*Simulated flared root canals, open apex.*	Flare: White-Yellow-Red-Blue RelyX™ post drills. Open apex: Peeso reamer size #1 - #6	(FRC) post (RelyX™, 3 M Oral Care, St. Paul, MN, United States)
3 (*n* = 16)	*Simulated flared root canal, closed apex*	Flare: White-Yellow-Red-Blue RelyX™ post drills.	(FRC) post (RelyX™, 3 M Oral Care, St. Paul, MN, United States)
4 (*n* = 16)	*Simulated flared root canals, open apex.*	Flare: White-Yellow-Red-Blue RelyX™ post drills. Open apex: Peeso reamer size #1 - #6	Customized (FRC) posts (RelyX™, 3 M Oral Care, St. Paul, MN, United States) relined with flowable E-glass discontinuous short fiber-reinforced composite resin (everX Flow™ Bulk, GC, Tokyo, Japan)
5 (*n* = 16)	*Simulated flared root canal, closed apex*	Flare: White-Yellow-Red-Blue RelyX™ post drills.	Customized (FRC) posts (RelyX™, 3 M Oral Care, St. Paul, MN, United States) relined with flowable E-glass discontinuous short fiber-reinforced composite resin (everX Flow™ Bulk, GC, Tokyo, Japan)

A standard, prefabricated RelyX™ fiber post (3 M Oral Care, St. Paul, MN, United States) size #1 (Yellow) was tried in the canal to ensure the fit and resistance of the post following post-space preparation for Group 1. On the other hand, for groups 2–5, RelyX™ fiber post size #1 (Yellow) was tried in the canal and checked for loose fitment. In all groups the posts were removed and cleaned with isopropyl alcohol and air-dried. Subsequently, Scotchbond™ universal adhesive (3 M Oral Care, St. Paul, MN, United States) with a combined formulation of adhesive resin and silane was applied over the posts and scrubbed for 20 s according to manufacturer’s instructions, followed by air thinning and light-cured for 40 s using the BluePhase Style (Ivoclar vivadent, Germany) light curing unit, at an output intensity of 1200 mW/cm^2^. In Groups 4 and 5, the radicular dentin was coated with glycerin-separating agent using a thin micro brush. EverX Flow™ (GC, Tokyo, Japan) resin composite was applied around the posts. The posts were then inserted into the root canals and light-cured for 40 s from the occlusal aspect. The anatomically-customized posts were removed, light-cured for 20 s from all sides, cleaned with water to remove any residues of the separating agent, and dried.

All the prepared canals were irrigated with water and dried with paper points. RelyX™ Unicem self-adhesive universal Resin Cement (3 M Oral Care, St. Paul, MN, United States) was introduced into the canals using the supplied plastic intra-canal tips. After placement of the posts (Groups 1–3) or the anatomically customized posts (Groups 4 and 5), the excess cement was removed, and light cured for 40 s.

Standard pre-formed celluloid crowns (3 M Oral Care, St. Paul, MN, United States) with premolar shape were used as molds for core built up using Filtek™ Bulk Fill restorative composite (3 M Oral Care, St. Paul, MN, United States). The coronal dentin surfaces were treated with Scotchbond™ Universal adhesive (3 M Oral Care, St. Paul, MN, United States) for 20 s and light cured for 10 s. The molds were filled with Filtek™ Bulk Fill restorative composite (3 M Oral Care, St. Paul, MN, United States) and applied onto the restored roots followed by light curing for 40 s. The molds were removed, and the composite cores were further light cured for 20 s from all aspects. A radiometer was used after finishing each group of samples to assess the accuracy of irradiance and power of the used light-curing unit.

### Artificial aging

All groups were subjected to artificial aging (thermo-mechanical cycling) in a computer-controlled chewing simulator (SD Mechatronik CS-4.8, SD Mechatronic GmbH, Feldkirchen-Westerham, Germany). Specimens were subjected to 7500 thermal cycles between 5 and 55°C, with a dwell time of 30 s and a transition time of 6 s. Simultaneously, specimens were exposed to 500,000 cycles of mechanical loading with a dead weight of 5 kg and a frequency of 0.8 Hz (40 mm/s downward speed and 20 mm/s speed of lateral movement) through a steatite antagonist (Ø 4.0 mm) which hits the central fossa of the crown while sliding for a distance of 1.5 mm from the fossa toward the buccal cusp tip adapted from a previously described protocol [19]. The cyclic loading force was applied within the range of 17 and 44 N.

At regular intervals (every 100,000 cycles), the chewing simulator was stopped. The crowns and the exposed root surfaces were evaluated for chipping, large cracks and fractures. After completion of the aging process, the samples were examined under a stereo microscope.

### Fracture resistance test

Following artificial aging, the survived samples were subjected to the fracture resistance test which was conducted following a protocol mentioned in a previous study [20]. Each sample was placed in a universal testing machine at an angle of 45 degrees between the long axis of the tooth and the loading jig of the testing machine with a 5 kg load cell. Force was applied through a stainless-steel ball (2.0 mm in diameter) representing the antagonist tooth. Load was applied to the buccal slob of the palatal cusp at a cross head speed of 1.0 mm/min. Samples that failed and fractured during thermo-mechanical aging were treated as left-censored and were given a value of 0 N.

### Fracture pattern analysis

Fracture pattern analysis was performed through visual inspection under x3.5 magnification. The type of failure was classified following the descriptions by Vire, (1991) [21]:

- Type I: complete or partial debonding of the post and core without fracture (favorable failure).
- Type II: fracture of the post and/or core without fracture of the tooth (favorable failure).
- Type III: fracture of the post and core/tooth complex above the height of bone level simulation (favorable failure).
- Type IV: fracture of the post and core /tooth complex below the height of bone level simulation (catastrophic failure).

### Push-out bond strength test

The remaining 8 samples from each group (*n* = 8) were transversally sectioned using a low-speed diamond saw (IsoMet 1000, Buehler Ltd., IL, USA) under water cooling. Three standardized slabs measuring 1.5 mm thick per root were collected and designated as cervical, medium, and apical specimens. Each slab was positioned on a push-out jig. 50 N load was applied at a crosshead speed of 0.5 mm/min until the post was dislodged. The push-out pin (0.5 mm, 1.0 mm in diameter) was positioned in the center of the post surface without placing undue strain on the adjacent post space walls. The push-out bond strength (P) was calculated by dividing the load at failure (F) by the internal bonded surface area of the root canal (A) to be expressed in MPa.

$$\text{P}\hspace{0.17em}=\hspace{0.17em}\text{F}/\text{A}$$

$$\text{T}\text{o}\text{t}\text{a}\text{l}\, \text{S}\text{u}\text{r}\text{f}\text{a}\text{c}\text{e}\, \text{A}\text{r}\text{e}\text{a} \left(\text{A}\right) = {\uppi } (r1\hspace{0.17em}+\hspace{0.17em}r2)\sqrt{{(r1-r2)}^{2}+{h}^{2}}$$

*r*1: is the radius of the post from the upper part of the specimen, *r*2: is the radius of the post from the lower part of the specimen, *h*: is the height of the specimen.

After debonding, the specimens were sputter coated with 100 Å Gold-Palladium (EMS 7620 Mini Sputter Coater, Hatfield, Pennsylvania, United States) and examined under a scanning electron microscope (SEM; VEGA3 XM–TESCAN, Kohoutovice, Czech Republic).

The failure mode was classified into four categories:

1. Type I: Adhesive failure between fiber post and resin cement;
2. Type II: Adhesive failure between fiber post and resin composite;
3. Type III: Adhesive failure between the resin composite and resin cement;
4. Type IV: Adhesive failure between resin cement and root dentin.

### Statistical analysis

All data were entered and analysed using the IBM SPSS version 26.0 (SPSS Inc, Chicago, IL, USA). The Kolmogorov-Smirnov test indicated that the data were normally distributed. For artificial aging, a Kaplan-Meier survival analysis test was performed, followed by a post-hoc log rank test for pairwise comparison. A One-way ANOVA was used to evaluate the data for fracture resistance and a post-hoc Tukey’s HSD test. To assess the failure patterns observed, Chi-square test was conducted. However, due to the assumption of using a Pearson Chi-square was not fulfilled, Fisher’s Exact was utilised. For push-out bond strength test, a two-way ANOVA was used to examine the effect of the restorative group and the variable root thirds on the push-out bond strength. A p-value of less than < 0.05 was considered to be statistically significant.

## Results

### Artificial aging

**Fig. 2:**
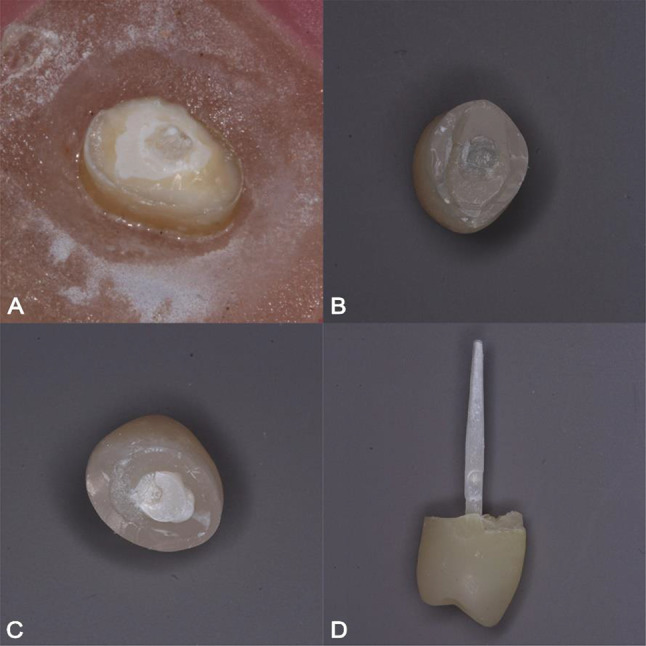
Different failure patterns after artificial aging. (**A**–**C**) Complete core fracture. (**D**) Complete debonding of the post/core complex

The mean survival times of the experimental groups are presented in Table 2. The different failure types after thermo-mechanical aging are displayed in Fig. 2.

**Table 2:** Mean survival time of the different experimental groups

Group	Mean survival time (cycles) (95% CI*)
Group1	487500.0 (464582.35, 510417.65)
Group2	262500.0 (158916.85, 366083.15)
Group3	337500.0 (246239.56, 428760.45)
Group4	475000.0 (444993.75, 505006.24)
Group5	475000.0 (444993.75, 505006.25)
Overall	407500.0 (366863.96, 448136.04)

*95% confidence interval

**Fig. 3:**
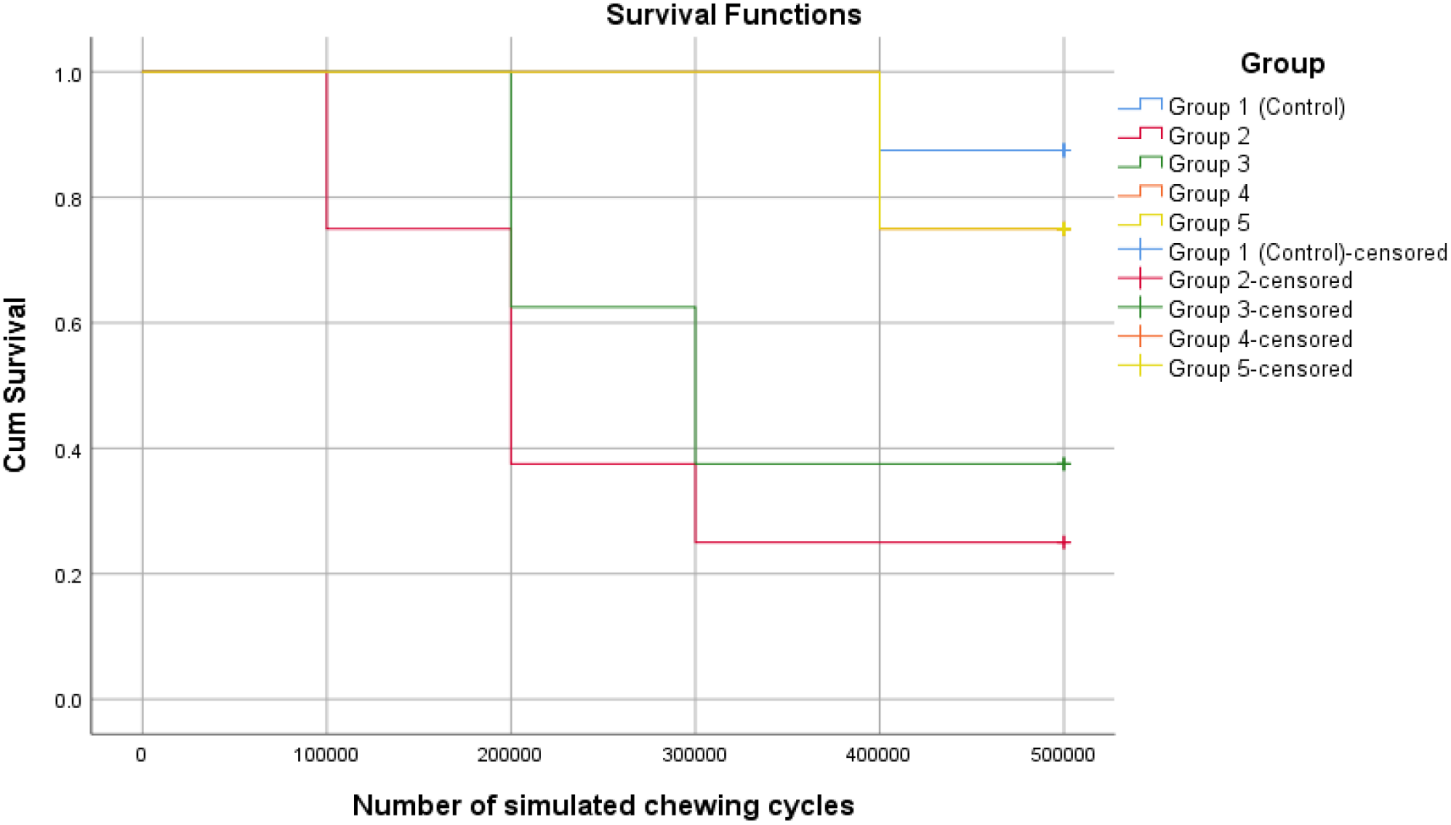
Thermo-mechanical aging survival curve (Kaplan-Meier survival analysis) for experimental groups loaded with 50 N

The Kaplan-Meier survival graphs for the thermo-mechanical aging test of simulated normal chewing force (50 N) of different restorative groups are displayed in Fig. 3. Table 3 presents the p-values for post-hoc pairwise log-rank comparisons among the experimental groups after the thermo-mechanical aging test.

**Table 3:** Results for post-hoc pairwise log-rank comparisons among the experimental groups following thermo-mechanical aging test

Restorative technique used		Group1		Group2		Group3		Group4		Group5	
		Chi-Square	Sig.	Chi-Square	Sig.	Chi-Square	Sig.	Chi-Square	Sig.	Chi-Square	Sig.
Log Rank (Mantel-Cox)	Group1			7.238	0.007	4.712	0.030	0.385	0.535	0.385	0.535
	Group2	7.238	0.007			0.907	0.341	5.888	0.015	5.888	0.015
	Group3	4.712	0.030	0.907	0.341			3.314	0.069	3.314	0.069
	Group4	0.385	0.535	5.888	0.015	3.314	0.069			0.000	1.000
	Group5	0.385	0.535	5.888	0.015	3.314	0.069	0.000	1.000		

*p - value* is significant at the 0.05 level (*p* < 0.05)

The chewing simulator was interrupted at regular intervals of 100,000 to check the condition of the samples. Failure in the form of complete core fracture at the level of cemento-enamel junction was seen in all experimental groups at different intervals of 100,000 cycles, 300,000 cycles and 400,000 cycles. In addition, complete debonding of the post and core complex was seen in Group 2 and Group 3 at 100,00 cycles and 300,000 cycles.

Pairwise log-rank comparisons revealed that there was no statistically significant difference between Group 1 (control group), Group 5 and Group 4 as well as between Group 2 and Group 3 (*p* > 0.05). Group 2 and Group 3 showed a statistically significant lower survival rate compared to all other groups (Table 3).

### Fracture resistance and fracture pattern

One-way ANOVA test revealed a statistically significant difference in the fracture resistance values between the different groups (Table 4) (*p* < 0.05).

**Table 4:** Comparison of mean fracture resistance between groups using One-way ANOVA test

Group	n	Fracture Resistance (N)	F- statistics (df)	p-value
		Mean (SD)		
Group 1	7	1201.71 (39.186) ^A^	292.569 (4)	< 0.001^*^
Group 2	2	758.00 (52.326) ^B^		
Group 3	4	882.00 (31.113) ^B^		
Group 4	6	1265.67 (54.680) ^A^		
Group 5	6	1795.83 (60.589) ^C^		

df: degree of freedom x*p < 0.05 is significant

Values with similar uppercase superscript letters indicate no statistically significant difference (*p* > 0.05)

**Fig. 4:**
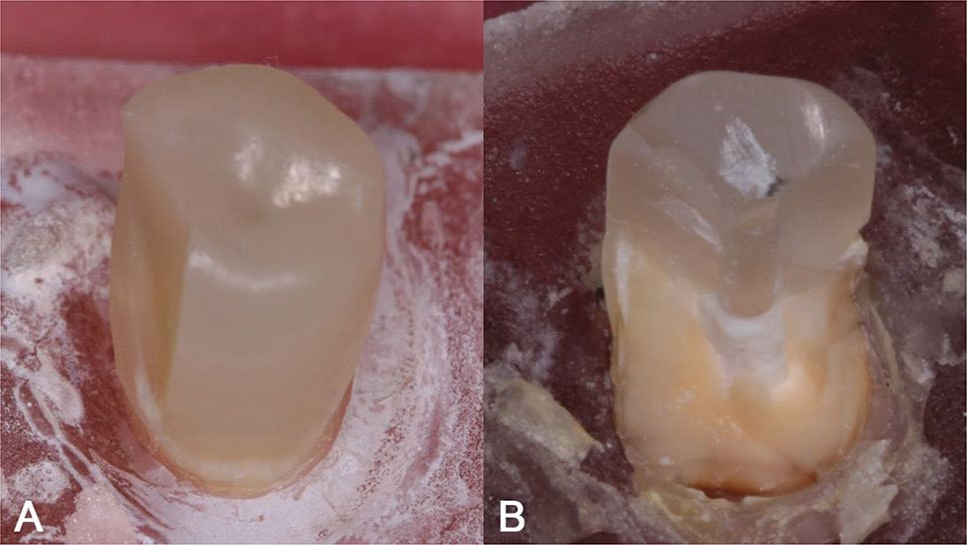
Fracture patterns following load to failure test. (**A**) Favorable fracture. (**B**) Unfavorable (catastrophic) fracture

In comparison to the other groups, Group 5 had the highest fracture resistance (1796 N), which was statistically significant (*p* < 0.05), while Group 2 showed the lowest mean fracture resistance (758 N) and was significantly lower compared to other groups. There was no statistically significant difference between Group 1 (control group) and Group 4. Both groups restored with the anatomically customized FRC post presented higher mean fracture resistance compared to groups restored with standard FRC post alone. Regarding the mode of failure observed, Fisher’s Exact test was conducted to evaluate fracture patterns among the experimental groups (Table 5). Groups restored with a customized FRC post (Group 4 and 5) presented favorable fractures, whereas the remaining groups including the control groups presented unfavorable fracture patterns. Several types of failures observed in the specimens of different experimental groups are shown in Fig. 4.

**Table 5:** Comparison of fracture pattern after artificial aging and fracture resistance tests between groups using Fisher’s Exact test

Group	n	Fracture pattern		Value	p-value
		Favorable n (%)	Unfavorable n (%)		
Group1	8	2 (25.00)	6 (75.00)	10.464	0.036^*^
Group2	8	4 (50.00)	4 (50.00)		
Group3	8	3 (37.50)	5 (62.50)		
Group4	8	7 (87.50)	1 (12.50)		
Group5	8	7 (87.50)	1 (12.50)		

*p < 0.05 is significant

### Push out bond strength

None of the specimens was found to have any artifacts or distortion following the sectioning procedure by the precision saw. Results of the Two-Way ANOVA indicated that both main effects which are the restorative group, the root third and their interaction are statistically highly significant (*p* < 0.001) (Table 6). The mean push-out bond strength values are displayed in Table 6.

**Table 6:** Mean values for the push-out bond strength (MPa) by restorative group, root third and their interaction

a. Restorative Group	**Group 1**	**Group 2**	**Group 3**	**Group 4**	**Group 5**
b. Root third	10.13 (2.33) ^A^	7.76 (2.34) ^B^	8.77 (1.99) ^C^	11.95 (1.48) ^D^	13.11 (2.15) ^E^
	**Cervical**	**Middle**	**Apical**		
c. Restorative group X Root third	12.25 (2.37) ^A^	11.00 (1.46) ^B^	7.77 (2.45) ^C^		
	**Group 1**	**Group 2**	**Group 3**	**Group 4**	**Group 5**
Cervical	12.20 (0.78) ^A^	9.48 (0.40) ^B^	10.25 (0.79) ^B^	13.59 (0.58) ^C^	15.73 0.64) ^D^
Middle	11.08 (0.80) ^A^	9.21 (0.39) ^B^	9.89 (0.70) ^B^	12.05 0.43) ^C^	12.82 0.64) ^C^
Apical	7.11 (0.52) ^A^	4.60 (0.60) ^B^	6.16 (0.36) ^C^	10.21 0.41) ^D^	10.79 0.46) ^D^

Within each raw, values with similar uppercase superscript letters indicate no statistically significant difference (*p* > 0.05)

In the restorative group comparison (Table 6), Group 5 scored the highest mean push-out bond strength compared to all other groups. Groups with anatomically customized FRC posts (Group 4 and Group 5) demonstrated significantly higher bond strength (*p* < 0.001) than groups with standard FRC posts (Group 2 and Group 3) and Group 1 (control). Regarding the different root thirds comparison (Table 6), the overall bond strength values was highest at the cervical third, followed by the middle and apical third. The differences between the variable root thirds were statistically significant (*p* < 0.001).

**Fig. 5:**
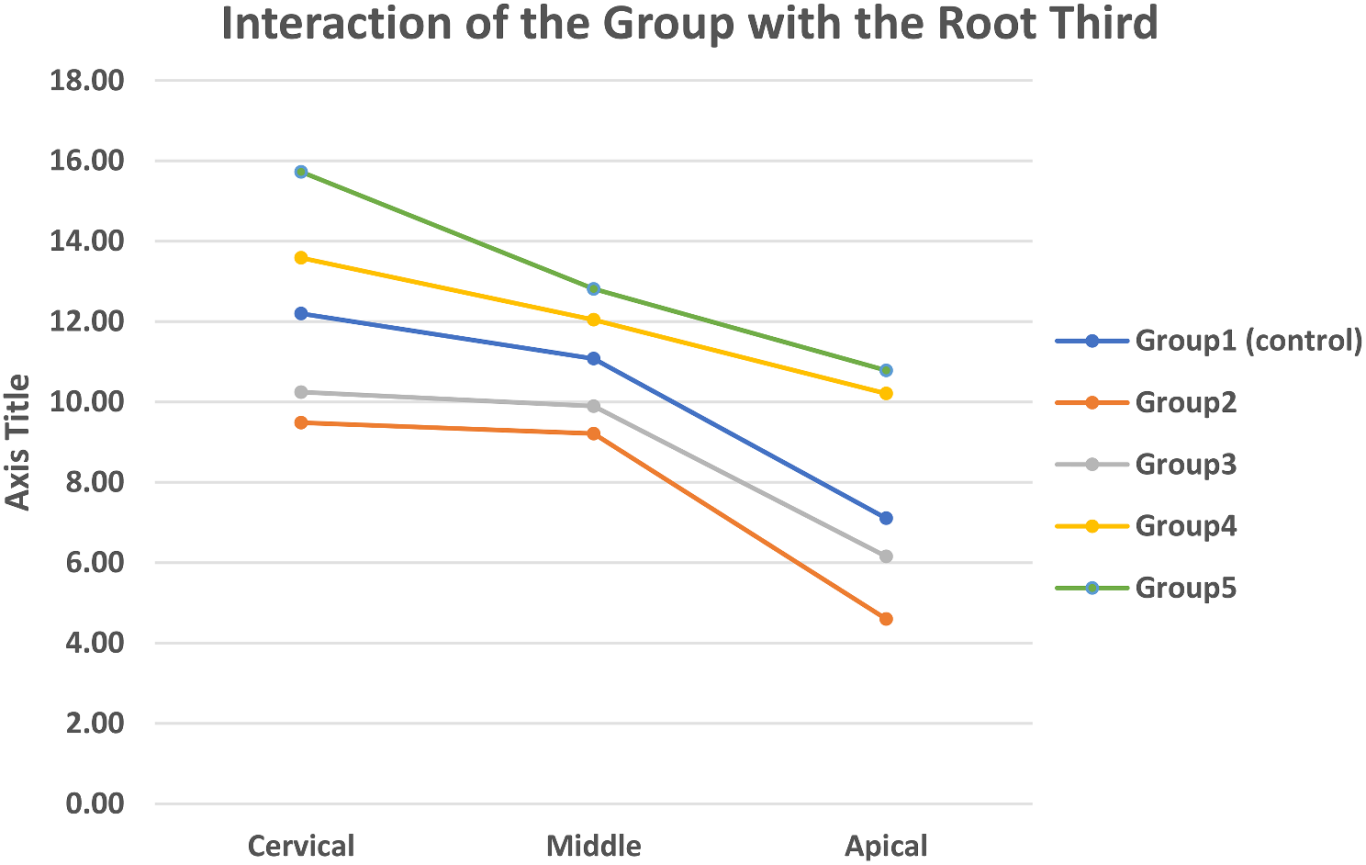
Interaction between the mean push-out bond strength of the restorative group and the root third

The mean bond strength values for each root third of the different restorative groups are also shown in Table 6. At all root thirds (cervical, middle, and apical), groups restored with anatomically customized FRC post (Group 4 and 5) displayed significantly higher bond strength values (*p* < 0.001) than both Group 1 (control group) as well as groups restored with standard FRC post (Group 2 and 3) Fig. 5.

**Fig. 6:**
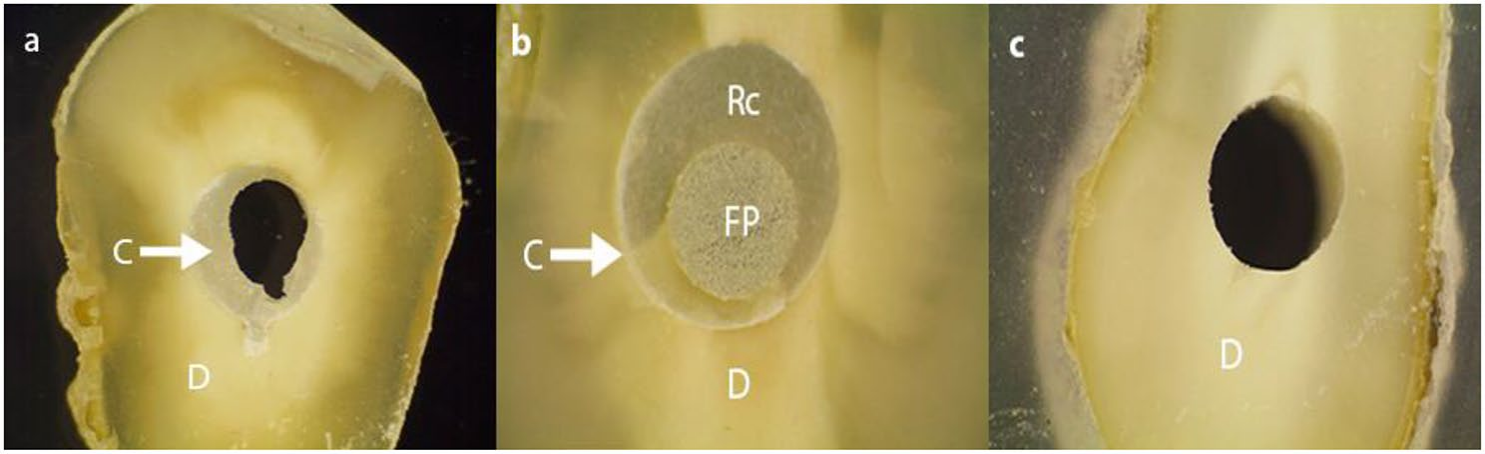
Different failure modes as seen by stereomicroscope. (**a**) Type I failure magnification of 5x. (**b**) Type II failure magnification 15x. (**c**) Type IV failure magnification 5x. (D, Dentin; C, Resin Cement; FP, Fiber Post; Rc, EverX Resin Composite)

**Fig. 7:**
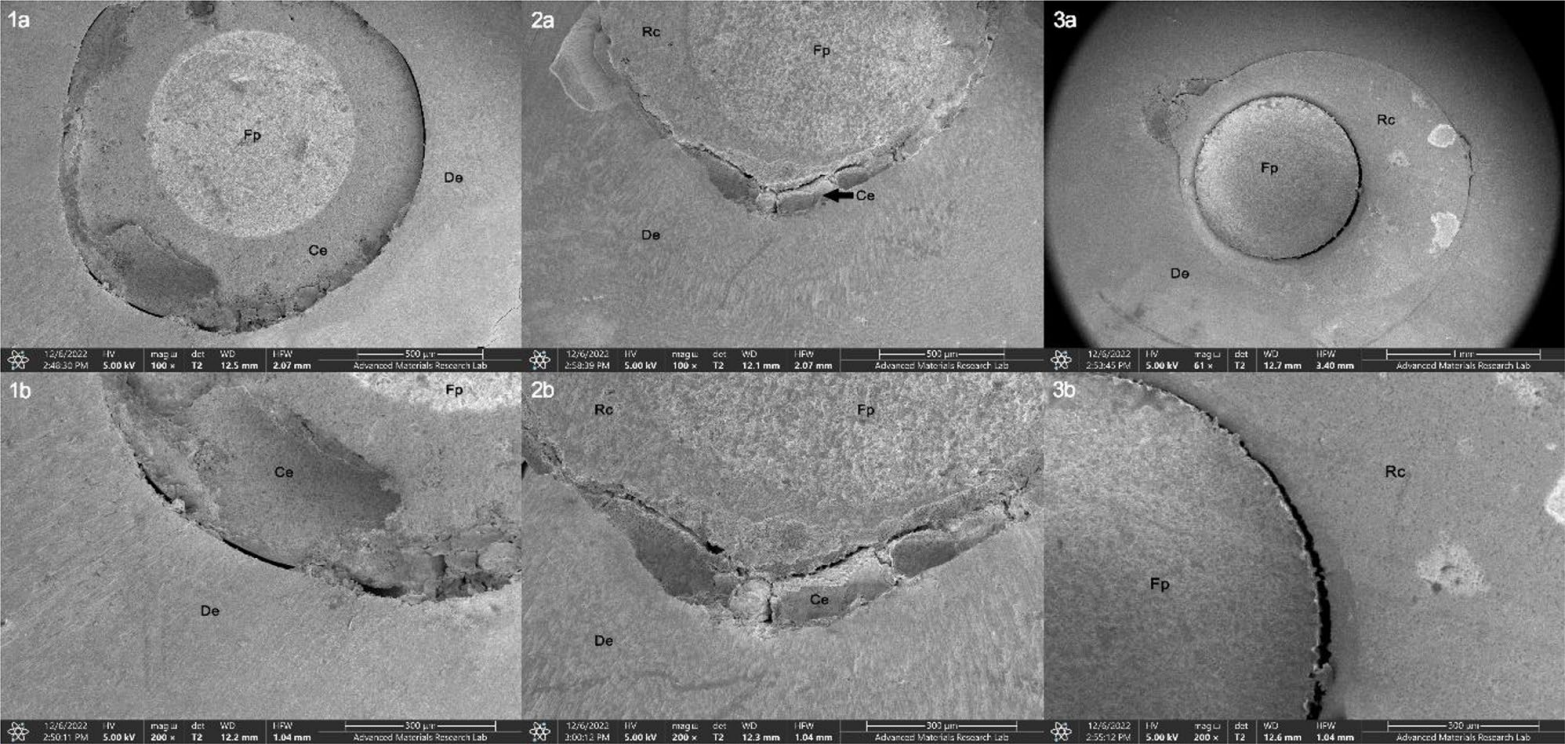
Representative SEM microimages for the tested specimens under x100 and x200 showing different adhesive failures: (**1a**, **b**) between resin cement and dentin (Type IV) in a sample restored with standard FRC post, (**2a**, **b**) between resin cement and resin composite (Type III) in a sample restored with anatomically customized FRC post, (**3a**, **b**) between fiber post and resin composite (Type II) in a sample restored with anatomically customized FRC post. (De, Dentin; Ce, Resin Cement; Rc, Resin composite Fp, Fiber Post)

Analysis of the mode of failure for the debonded samples by stereomicroscope and SEM revealed that the most frequently observed failure occurred between the resin cement and radicular dentin (Type IV) for all the groups, followed by failure between the fiber post and resin cement (Type I) as shown in Table 7. Different types of failures displayed in Fig. 6 (a–c) were captured by stereomicroscope while those displayed in Fig. 7 were captured by SEM.

**Table 7:** Distribution of failure modes among experimental groups

Group	Mode of failure			
	Type I n (%)	Type II n (%)	Type III n (%)	Type IV n (%)
Group 1	7 (29.2)	0	0	17 (70.8)
Group 2	10 (41.7)	0	0	14 (58.3)
Group 3	8 (33.3)	0	0	16 (66.7)
Group 4	4 (16.7)	3 (12.5)	4 (16.7)	13 (54.2)
Group 5	2 (8.3)	3 (12.5)	4 (16.7)	15 (62.5)

## Discussion

The results of the current study showed that there was no statistically significant difference in the survival rate between groups restored with anatomically customized FRC posts (Group 4 and 5) and Group 1 (control) following thermo-mechanical aging (*p* > 0.05). Groups restored with standard FRC posts (Group 2 and 3) showed significantly lower survival rate compared to Group 1 (control) and groups restored with customized FRC posts (Group 4 and 5) (*p* < 0.05). In addition, statistically significant difference in the fracture resistance and failure patterns were found among the experimental groups (*p* < 0.05). Thus, the first null hypothesis had to be rejected.

### Artificial aging

Two types of failure were noted in the samples submitted to thermo-mechanical aging. Different distribution of these failures was evident among the experimental groups, affecting mainly Group 2 and Group 3 at the early periods of chewing simulation (100,000–200,000). Meanwhile, failure of samples from the remaining groups (Group 1, 4 and 5) in the form of complete core and post fracture was observed at later periods of chewing simulation (400,000–500,000).

The higher incidence of complete debonding of post and core complex in Group 2 and 3 can be explained by the mismatch in dimensions and geometry of the fiber posts and the simulated flared root canals, which was more evident in Group 2 with simulated open apex as the walls were prepared parallel without any taper. In a previous study carried out by the same research group, anatomically customized FRC posts in flared canals with closed or open apex presented significantly higher adaptation and fitment to radicular walls than in samples with flared canals and closed or open apex restored with standard FRC posts [9]. The inferior adaptation in Group 2 and Group 3 might have led to the presence of an excessively thick cement layer occupying majority of the root canal space in the coronal, middle and apical region. The cement layer zone is considered as a weak junction between the fiber post and radicular dentin where voids and gaps are commonly seen [22]. In addition, complete debonding can be attributed to the increased polymerization shrinkage stresses due to the excessive volume of resin cement used to fill the wide gap at the post-dentin interface in such a high C-factor cavity (root canal space), ultimately leading to debonding of the post [5, 23, 24]. Previous studies reported improved bond strength values as the cement layer thickness decreased and vice versa [25, 26]. Moreover, the presence of a highly crosslinked polymer matrix with high degree of conversion found in conventional FRC posts has been reported to result in poor adhesion between prefabricate FRC posts and other resin-based materials or the tooth structure [27].

### Fracture resistance and failure pattern analysis

All failures encountered during the artificial aging test were given a fracture load value of “0 N” to account for the effect of artificial aging in statistical evaluation of fracture resistance data. Normality of data distribution was also confirmed to assess the reliability of conducting a parametric test, and data was normally distributed with assumption of equal variance. Various aspects can explain the higher fracture resistance and the more favorable fracture pattern observed with the groups restored with customized FRC posts. In the current study anatomically customized posts, fabricated by relining continuous unidirectional FRC posts with bulk-fill flowable SFRC, were used to restore the intraradicular region in Group 4 and Group 5. As mentioned earlier, relining the FRC posts improved the intimate contact and adaptation between these customized post and radicular dentin walls, leading to more frictional retention and a more homogenous and thinner resin cement layer. Therefore, detrimental factors such as polymerization shrinkage stress, formation of voids and bubbles in cement layer are expected to reduce, minimizing the chances of debonding and enhancing the fracture resistance. Similar findings regarding the improved internal adaptation and intimate contact of anatomically customized FRC posts compared to non-customized fiber posts in wide compromised root canals, and their effect on increasing the mean fracture strength was reported by a few other studies [8, 16].

In addition to the verified advantages of bulk-fill flowable resin composites present over conventional resin composites and resin cements such as lower polymerization shrinkage stress and improved light transmission that allows bulk placement even in cavities with high C-factor [28, 29], relining FRC posts with the recently introduced bulk-fill flowable SFRC, with its unique composition and properties, may have added further benefits in the restoration of weak ETT with compromised canal configurations, following the biomimetic approach. SFRC was proved to have superior fracture toughness, flexural strength and modulus compared to conventional resin composite. Moreover, SFRC has specific properties such as termination of crack propagation, lower polymerization shrinkage stress, reduced micro gap formation and microleakage compared to other available bulk-fill and conventional resin composites [10, 11].

The semi-Interpenetrating matrix (Semi-IPN) of the flowable SFRC used to reline the standard FRC contains linear and cross-linked polymer phases. The linear phase provides readily available substrate for excellent bonding to posts, cements and dentin compared to standard FRC [10, 27]. Furthermore, the volume of fibers in the critical cervical region is maximized by relining conventional FRC posts with flowable SFRC. Simultaneously, the resin cement thickness was minimized as recommended by authors in previous study [30]. The cement layer in Group 4 and group 5 was replaced with a stronger material which not only has lower polymerization shrinkage, but also induces less stress as its polymerization takes place prior to cementation. Additionally, the presence of randomly oriented short fibers has an important role in transferring stresses from the polymer matrix to the fibers which is responsible for the reinforcement effect seen when SFRC is used [31]. Therefore, it can be postulated that the unique composition of SFRC and its beneficial influence on the material’s properties participated in the shift of the fracture pattern in Group 4 and Group 5 to a more favorable pattern in the form of partial core fracture, limited to the level of cemento-enamel junction. On the contrary, according to the findings of the current study, it was evident that the use of conventional FRC post alone in flared compromised root canals resulted in catastrophic unfavorable fracture below the level of simulated crestal bone.

### Push-out bond strength test and mode of failure analysis

The second null hypothesis was also rejected since the types of posts used affected the bond strength and significant differences were found between the groups. This is in agreement to the findings of Fantin et al. and Alves et al., who reported higher bond strength values in groups restored with customized posts compared to non-customized posts following a push-out bond strength test [6, 32].

The main advantage of relining prefabricated FRC posts is reducing the resin cement layer thickness when used in compromised wide root canals, which is associated with reduced defect formation such as voids and bubbles which are considered areas of stress formation and contribute to debonding [6, 33]. Additional benefits include increased mechanical friction resistance [34], improved bond strength [22, 25, 26] and reduced polymerization shrinkage stresses in the low thickness film of cement [4, 6, 24, 32].

Improved intracanal adaptation was confirmed with the anatomically customized FRC posts [9]. This means that the excessive space around the post in a flared root canal is being occupied by the relining resin composite material with sufficient curing, mechanical and physical properties instead of the weak resin cement layer. The initial curing of the relining composite is enhanced by delivery of abundant amount of light through the prefabricated post and randomly oriented short fibers with further curing outside the root canal. In addition, it will be accompanied upon cementation by an increase in hydraulic pressure exerted on the cement material. This pressure leads to increase the number of dentinal tubules filled with resin due to better penetration, as well as reduced chance for air entrapment and bubbles formation [35, 36]. As a result, the higher bond strength evident in groups restored with anatomically customized FRC posts can be attributed to the advantages of the relining technique.

When comparing the bond strength values of different root parts, the cervical and middle root thirds were always having higher value of bond strength than the apical third in all groups. The results are due to the reduced thickness of the resin cement, and higher fiber volumes with better stress distribution in these regions. Lower values in the apical third can be attributed to many factors such as inadequate polymerization with reduced degree of conversion of resin monomers at this deep area [7, 37]. Additionally, it can be explained by the increased volume of resin cement found apically, due to the reduced taper of the fiber post towards the apical region, as well as the difficulty in adhesion encountered in apical radicular dentin as a result of substrate composition in the form of decreased number, density and diameter of dentinal tubules [38].

In the current study, a significantly higher bond strength values in the apical third of Group 5 and Group 4 was found compared to other groups. The combined use of a prefabricated FRC post with the translucent flowable SFRC results in improved light transmission and ultimately light polymerization for the resin cement in the apical region. The prefabricated FRC post acts as an efficient light transmitting rod and the translucent nature of the flowable SFRC with the composing randomly oriented short fibers aid in delivering light over longer distances. This in turn improves the polymerization of the resin cement at the deep apical region, contributing to improved bond strength values. Similar effect was also reported in previous studies [12, 39].

A combined evaluation of the failure mode using a stereomicroscope in addition to SEM was done to obtain higher magnification and more detailed images of the specimens in cases where the failure was not clear or unidentifiable by the stereomicroscope. Multiple adhesive failures were evident at post-cement interface (Type I), post-composite interface (Type II), composite-cement interface (Type III) and cement-dentin interface (Type IV) with different percentages as shown in Table 7. Type IV failure was the most prevalent among the control, non-relined and relined groups. Bonding of resin to radicular dentin is challenging and limited by anatomical, histological and procedural factors [38]. The inherent polymerization contraction of resin-based materials generates forces that can exceed the bond strength of the cement to dentin. This is particularly high where a thick resin cement layer is present such as in flared canals with non-relined fiber posts. Higher configuration factor (C-factor) is present due to the root canal morphology and may exceed 200 score leading to extremely unfavorable condition for restorative procedures as less flow or material deformation is allowed for stress relief [40]. Other factors such as the smear layer resulting from post space preparation, presence of debris that was not completely removed by root canal instrumentation and collagen degradation by host derived matrix metalloproteinases (MMPs) can influence the adhesion at the cement-dentin interface [41]. Moreover, failure at the post-cement interface (Type I) was also highly frequent in the control and non-relined groups (29.2−41.7%). Although a universal adhesive with silane coupling agent (Scotchbond universal) has been applied to the external surface of the fiber post prior to cementation to improve the wetting of the post with resin cement [42], it did not seem to enhance the bonding quality to the resin cement. Compared to the relined groups (Groups 4 and 5), the post-composite bond was greater, shifting the failure to the weaker cement-dentin interface (Type IV) mostly in these groups, as the presence of the linear polymer phase in flowable SFRC was found to improve the material’s bond strength to other substrates [10, 27].

In agreement with previous recommendations from other authors, flowable SFRC presents a better option to replace missing dentin, compared to conventional particle-filled resin composites and dual cure core build up materials, as the formers have significantly higher fracture toughness and enhanced mechanical behaviour [12, 30–32, 43]. Consequently, due to all the aforementioned benefits of flowable SFRC that was supported by the findings of the current study, the SFRC can be considered the material of choice for relining FRC posts in compromised root canals.

The present study investigated different restorative techniques on small scale population sample of premolar teeth, therefore is subjected to inherent limitations associated with such design and clinical extrapolation of the results should be done cautiously. These limitations include small number of samples due to unavailability of teeth with similar dimensions. In addition, only one type of human teeth was included in the study (premolar teeth). Furthermore, a single type of cement was used for cementation in all groups. It is recommended to compare the effects of different types of cements (adhesive, self-adhesive resin cements) on the mechanical properties and bonding performance of FRC posts relined with SFRC.

## Conclusion

Within the limitations of this study, restoring ETT with compromised root canals using FRC posts anatomically customized with bulk-fill flowable SFRC resulted in higher survival rate compared to standard non-relined prefabricated FRC posts. Superior fracture resistance and favorable failure patterns were attained when utilizing FRC materials that combine both discontinuous short and continuous long fibers, and the tooth remains restorable after failure. In addition, the exclusive composition and properties of the bulk-fill flowable SFRC relining material increased bond strength values of the anatomically customized restoration to radicular dentin, including the critical apical region.

## Data Availability

The datasets used and/or analyzed during the current study are available from the corresponding authors on reasonable request.
